# The Promise and Challenge of State-Based Long-Term Care Insurance

**DOI:** 10.1093/geroni/igaf122.1662

**Published:** 2025-12-31

**Authors:** Alicia Munell

**Affiliations:** Boston College, Chestnut Hill, Massachusetts, United States

## Abstract

The cost of Long-Term Care is unaffordable for most people. Paid home care for an individual with significant needs can easily exceed $35,000 per year which is more than the total annual income of about half of older Americans and more than the total net worth for about one-fifth. The state of Washington launched We Care, a publicly funded long-term care insurance policy in 2019. The first benefits will be paid in 2026. This presentation will address some of the challenges that the designers of the program faced, for example managing adverse selection, improving fairness and allowing some people to opt-out. The Washington program has some important lessons for other states that are exploring similar policies, but state variation in tax rates and benefit levels raise new questions about what level of benefit is adequate to support people who need long-term care.

